# An Effective and Efficient Approach for 3D Recovery of Human Motion Capture Data

**DOI:** 10.3390/s23073664

**Published:** 2023-03-31

**Authors:** Hashim Yasin, Saba Ghani, Björn Krüger

**Affiliations:** 1 Department of Computer Science, National University of Computer and Emerging Sciences, Islamabad 44000, Pakistan; saba.ghani@nu.edu.pk; 2 Faculty of Information, Media and Electrical Engineering, Institute of Media and Imaging Technology, TH Köln—University of Applied Sciences, 50679 Köln, Germany; bjoern.krueger@th-koeln.de

**Keywords:** 3D recovery, human motion capture, missing joints or markers, kd-tree, K-nearest neighbors, optimization, GPU

## Abstract

In this work, we propose a novel data-driven approach to recover missing or corrupted motion capture data, either in the form of 3D skeleton joints or 3D marker trajectories. We construct a knowledge-base that contains prior existing knowledge, which helps us to make it possible to infer missing or corrupted information of the motion capture data. We then build a *k*d-tree in parallel fashion on the GPU for fast search and retrieval of this already available knowledge in the form of nearest neighbors from the knowledge-base efficiently. We exploit the concept of histograms to organize the data and use an off-the-shelf radix sort algorithm to sort the keys within a single processor of GPU. We query the motion missing joints or markers, and as a result, we fetch a fixed number of nearest neighbors for the given input query motion. We employ an objective function with multiple error terms that substantially recover 3D joints or marker trajectories in parallel on the GPU. We perform comprehensive experiments to evaluate our approach quantitatively and qualitatively on publicly available motion capture datasets, namely CMU and HDM05. From the results, it is observed that the recovery of *boxing*, *jumptwist*, *run*, *martial arts*, *salsa*, and *acrobatic* motion sequences works best, while the recovery of motion sequences of *kicking* and *jumping* results in slightly larger errors. However, on average, our approach executes outstanding results. Generally, our approach outperforms all the competing state-of-the-art methods in the most test cases with different action sequences and executes reliable results with minimal errors and without any user interaction.

## 1. Introduction

The capturing, synthesis, and analysis of human motions have been very active research areas in computer graphics and animation for the last few decades. Motion is always considered an essential hint or cue for analyzing and understanding human actions, activities, and behaviors. Thus, the motion sequences are recorded in the format of 3D marker trajectories in an indoor studio-like environment. These 3D marker trajectories are further transformed into a 3D skeleton that is comprised of a number of 3D kinematic joints [1,2]. In general, the motion capture (MoCap) data are generated in 3D articulated movements of the connected joints of the human skeleton. The final MoCap data consist of spatiotemporal trajectories of human skeleton joints [3]. The recorded MoCap data are widely deployed in a variety of applications in order to execute natural-looking animations, i.e., in the generation of the animated characters in movies, video games, sports training, reality-based virtual applications, performance analysis of the players, human–computer interaction, biomechanics, and medical rehabilitation [4,5,6,7]. As a result, the demand for robust and accurate MoCap data is growing day by day.

Even with the sophisticated indoor professional-like environment and up-to-date software, the process involved in MoCap data acquisition is not mature enough to handle various artifacts and to produce exactly accurate motion captured data efficiently. The raw motion captured data are often corrupted due to the marker loss, noise, marker swaps, the lack of precise and proper equipment, joint occlusions, marker sheddings, etc. [5,8]. Consequently, all these factors hinder the production of accurate MoCap data. As a treatment, either the poses carrying incomplete markers information are entirely discarded, or intensive manual postprocessing is performed in order to cure the original data. The existing automated systems are not robust enough for postprocessing to detect and fix the corrupted or missing markers/joints properly. Eventually, professionally trained artists are hired to manually set the missing, corrupted, or noisy markers/joints, which is very labor-intensive and time-consuming. Such expensive treatments are highly required, as all applications are depending upon the accuracy and the precision of the MoCap data.

Moreover, due to the costly indoor studio-like environment, the reuse of the motion capture data or creating new realistic motion sequences from the existing motion clips are gaining significant importance [9]. Nevertheless, all these methods of reutilization of MoCap data require fast and efficient similarity search and retrieval techniques. MoCap data are generally high-dimensional, and due to its high dimensionality, there is a lack of efficient and fast similarity searching techniques, which need to be addressed. The lack of fast similarity search and retrieval in a high-dimensional MoCap dataset may be considered as the bottleneck in the performance of most of the MoCap data-dependent applications.

The first part of the research is dedicated to the parallel nearest neighbor search and retrieval from the MoCap dataset on GPU. Parallel processing provides a suitable solution for handling multiple queries simultaneously at a time and finding similar poses in a timely fashion as well. After retrieving the fixed number of K-nearest neighbors, we aim to utilize these similar poses to infer the 3D human poses and ultimately recover the missing markers/joints information of MoCap data. We propose an objective function with a variety of energy terms for recovery and reconstruction of the missing data. Our proposed approach is a frame-by-frame data-driven approach in order to recover missing 3D joints/markers from the given sequence of the 3D MoCap data that may be acquired through any sensor system such as magnetic, mechanical, inertial, optic, non-optic sensors, Kinect (depth) sensors, images/videos or hybrid systems.

The paper is organized as follows: Section 2 provides the details of the literature review and the state-of-the-art approaches that deal with the search and retrieval of nearest neighbors and the recovery of missing or corrupted 3D MoCap data. Section 3 demonstrates all the steps involved in our proposed pipeline. Section 4 explores the details of experiments, the comparisons with other state-of-the-art methods, and the evaluations (quantitative and qualitative), along with the conclusive remarks. In the end, we describe the limitations of our approach in Section 5 and conclude our work in Section 6. We also discuss a few future directions in Section 6.

## 2. Related Work

In this section, we discuss the related research works and techniques in the area of the fast searching and then retrieval of K-nearest neighbors from the MoCap dataset and 3D recovery of the motion sequences with missing/corrupted markers/joints in the MoCap dataset.

### 2.1. Approaches for Search and Retrieval

This subsection provides a review of the literature dealing with 3D human motion search and retrieval from MoCap dataset. There exist a variety of techniques in literature for search and retrieval of similar frames or poses from the dataset, i.e., clustering and segmentation based techniques [3,10,11,12,13], graph-based techniques [13,14,15,16], and methods based on data structures such as *k*d-tree [17,18,19,20,21,22].

Xiao et al. [10] propose quaternion and EMD-based nearest neighbors retrieval approach that depends on two steps: indexing and matching. They employ a K-means clustering strategy to categorize the features in the MoCap dataset. The matching part creates the distance matrix according to the clustering results. They deploy only 21 joints to represent the skeleton due to which the retrieval error is reduced. In our case, we are dealing with 40 to 50 markers, and the skeleton consists of 31 joints. Bernard et al. [11] propose MotionExplorer, a search system for the huge amount of motion capture data that exploit the hierarchical clustering method to generate the spatial aggregations of the MoCap dataset. The clustering in their approach is an interactive clustering since it requires user interactions in order to find useful cluster parameters as well as to interpret the acquired cluster. Vögele et al. [12] employ neighborhood graphs to partition the data and ultimately generate similar clusters based on similarity information collected from the neighborhood graph. In this way, they create motion primitives with semantic significance. Their method neither requires any assumptions about the input query sequences nor the user interaction. This method is extended further with kernel-based feature handling [13].

The recent works on graph-based retrieval from large motion capture databases deal with adaptive and weighted graphs [14,15,16]. The graphs are constructed adaptively according to the characteristics of the given motion. The graph kernel is then calculated by matching the two graphs [14]. Plantard et al. [15] developed a graphical structure called Filtered Pose Graph in order to evaluate the nodes that may contribute significantly to the process of pose reconstruction. In this way, they enhance system performance by improving the selected poses’ relevance and reducing the computation time. They provide such examples of the skeleton captured by Kinect that require reconstruction of only unreliable parts of the skeleton. For their system, it is mandatory to have information about which part of the skeleton is captured reliably and which part is recorded inaccurately. Panagiotakis et al. [16] perform an efficient graph-based searching strategy on the matrices of pairwise distances of poses of two sequences. An objective function further supports the result of the search.

The *k*d-tree-based local neighborhood searches, which lead to global similarity searches, were proposed [18]. The authors employ Euclidean distance measures to search the nearest neighbor from the developed *k*d-tree. The *k*d-tree-based neighborhood searches are extended further on 2D synthetic landmarks as well as 2D skeletons extracted from RGB images [17,23]. Yasin et al. [24] use *k*d-tree for the search and then retrieval of K-nearest neighbors (KNNs) which are employed further for human action classification and segmentation. The Surface Area Heuristic (SAH) *k*d-tree [19,20,21] have been constructed on GPUs in which the Surface Area Heuristic is calculated at each node to find the splitting plane axes instead of a median point. The computation of this cost function involves the costs for traversing to an inner node, intersecting triangles, and the surface areas of left/right/parent bounding boxes. All of these costs make these techniques applicable to ray tracing applications only. Hu et al. [25] propose an algorithm for *k*d-tree construction and KNNs search that utilizes GPUs’ Massive Parallel Architecture (MPA). Breadth-first search at each step of the *k*d-tree construction spawns a new thread for every node at an equal distance from the root node. Wehr and Radkowski [26] propose a parallel *k*d-tree construction algorithm with an adaptive strategy of split and sort. Only the construction algorithm has been presented without dealing with high-dimensional data. The *k*d-tree-based neighborhood search methods [17,18,21,23,25] require enough memory space to fit the *k*d-tree well into memory for proper running and execution of the system; otherwise, these methods do not scale well. Sedmidubsky et al. [27] discuss the existing techniques of the content-based management of the skeleton data. They also discuss a few future research directions for the management of large and diverse motion capture skeleton data. Lv et al. [28] propose a hash-based convolution neural network where they extract deep features using the VGG16 network. They introduce the hash layer to create the hash code and, as a result, CNN is fine-tuned. In the end, they retrieve the data using the learned hash codes.

### 2.2. Approaches for 3D MoCap Recovery

A bulk of methods have been proposed in order to recover missing information in the motion capture dataset. We categorize these approaches into two classes, the systems that deal with the recovery of missing markers and the techniques that deal with the recovery and reconstruction of missing joints.

#### 2.2.1. Approaches for 3D Recovery of Missing Markers

A number of approaches exist that deal with the 3D recovery of markers [29,30,31,32,33,34,35]. One of the early techniques is the interpolation method for estimating missing MoCap data [36]. Piazza et al. [29] use an extrapolation algorithm that is combined further with a constraint matrix for the recovery of missing markers from a pre-selected set of principal markers, after the prediction of immediate motion type, i.e., linear, circular, or both. Their prediction algorithm is independent of the human kinematic model. Baumann et al. [30] construct *k*d-tree for a structural organization of data and efficient retrieval of the nearest neighbors and then adapt an optimization method for recovery of the missing markers. In optimization, they also incorporate the velocity and acceleration of body joints to improve the system’s performance. This work focuses only on marker-based motion sequences and was tested on a very small data-set, only. The proposed optimization scheme is very simple, and does not consider any constraints such as bone lengths or other anatomical properties. Aristidou and Lasenby [31] recover 3D markers in occluded scenarios by employing the markers’ set of fixed relative positions. This information aid in an Unscented Kalman Filter (UKF) using a Variable Turn Model to predict the occluded markers automatically. The prediction is used further for joint localization and re-positioning of the joints through inverse kinematics. The proposed approach benefits from the assumption that the missing markers are visible to and captured by at least one camera, reducing the marker estimation error significantly.

Liu et al. [37,38] present data-driven, piecewise, and low-dimensional linear models in order to estimate and recover missing markers. The local linear model is identified for input query with missing markers, which fits the marker positions with the least square method. Peng et al. [32] propose an adaptive non-negative matrix factorization approach, which exploits the low-rank and the non-negative characteristics of MoCap data simultaneously in order to recover the incomplete block-based motion clips with missing markers, without considering any training priors and the user interference. The concept of mapping motions into blocks is implemented by utilizing the hierarchy of the defined body model. Wang et al. [33] present the data-driven approach for missing markers recovery based on the traditional sparse coding process. They design an objective function that comprises statistical and kinematical properties of motion capture data. They also exploit the Poselet model combined with the moving window to learn the motion dictionaries in parallel. Their approach does not work well when there is a motion with some sudden or abrupt change, but in our case, we introduce the smoothness error term which endorses the smoothness and avoids the jerkiness artifacts.

Kucherenko et al. [35] propose using neural network architecture for missing markers recovery. They perform experiments with two different artificial neural networks, i.e., Long Short-Term Memory (LSTM)-based and time-window-based. In window-based architecture, they deploy a fully connected artificial neural network, which is trained by the current input pose along with a range of previous time-steps. As a limitation, both methods, LSTM-based and time-window-based, are less stable and produce worse performance when the testing motion is not the part of the training data. In short, both methods do not contain generalization properties. On the other hand, our approach executes very good results even when the testing input query motion sequences are from the other MoCap dataset. Burke and Lasenby [39] combine the dynamic modeling-based tracking approaches with low-ranked matrix completion to solve the problem of missing markers recovery without having any prior knowledge of the marker positions. Their proposed methodology not only outperforms but also improves the position error. Hu et al. [34] introduce a novel approach in order to recover the incomplete MoCap data by exploiting a new matrix norm, called the truncated nuclear norm, that is based on the augmented Lagrange multiplier.

A Probabilistic Model Averaging (PMA) scheme for recovery of missing markers, proposed by Tits et al. [6], is a weighted combination of numerous recovery models based on interpolation, inter-correlations, and low-rank properties. It exploits the likelihood of the distances between the skeleton joints. In the reconstruction process, they impose skeleton constraints by introducing two heuristic algorithms. Their method is automatic, data-driven, self-sufficient, and independent from any pre-trained data model. As a limitation, the method does not deal efficiently with isolated markers as well as the markers when they are placed very close to each other. Moreover, PMA requires three present markers at least for reference points in order to evaluate the distance probabilities. Park et al. [40] apply PCA on marker positions in order to learn a statistical model. In low-dimensional space, the missing markers are recovered through the best fit of the available markers. This method is very effective when dense marker sets exist. Gløersen et al. [41] propose a reconstruction of corrupted marker trajectories, but their method is restricted to cyclic motions only, i.e., running or walking. A few strong assumptions are imposed, e.g., the existence of every marker for at least one-time step. The recovery of the markers in complex motions gives implausible results sometimes because of the linear basis models. Their approach is not perfect enough especially on the motion sequences with repeated movement patterns. Li et al. [42] propose a simple recovery method based on principal component analysis. They claim that their method has low time complexity and is numerically stable as well. As a limitation, their method does not deal with those markers that are missed in the whole motion sequence. Moreover, their method has limited ability to generalize with the addition of more training samples. Hai et al. [43] introduce the locally weighted PCA (LWPCA) regression method to recover the missing markers from motion capture data. The authors apply the weighted least square method combined with the sparsity constraints to PCA regression and improve the accuracy drastically. LWPCA executes high error on the joints-9,14 for the HDM05 mocap dataset and the joints-21,30,33 for the CMU mocap dataset.

#### 2.2.2. Approaches for 3D Recovery of Missing Joints

Much research has been carried out on the recovery of missing joints of 3D skeleton motion data [7,44,45,46,47,48]. Li et al. [44] propose a novel approach for 3D recovery of missing joints based on bidirectional recurrent autoencoder, with which they extract motion manifold grouped with smoothness and bone length constraints. Their method is neither action-dependent nor does it require the noise amplitude. Li et al. [45] propose a perceptual-based bidirectional recurrent autoencoder approach named BRA-P to refine the 3D skeleton motion data. They perform experiments on synthetic noise data as well as on raw motion data captured by MS Kinect. Xia et al. [7] present a tailored nonlinear matrix completion model, where multiple kernel learning processes have been exploited for combined learning of low-rank kernels. They transform the 3D motion data into Hilbert space, and then by employing an already learned kernel, they use low-rank matrix completion for 3D recovery of motions with missing joints. They also enforce the kinematic constraints to the recovered poses to maintain the human motions’ kinematic property, but the kinematic constraints are not sufficient to ensure the kinematic property. Moreover, their method is time-consuming and does not find a solution for the end-joint contact issue.

Lai et al. [46] exploit the low-rank matrix property for the recovery of MoCap data with some missing joints through the matrix completion method, namely Singular Value Thresholding (SVT), which was initially proposed by Cai et al. [49]. They deploy this simple first-order and iterative algorithm (SVT) and consider the missing joints’ recovery process as a problem of convex optimization. The authors apply soft-thresholding on singular values of the matrices efficiently to address the problem where the optimal solution has a low rank. The SVT method does not fit well when the missing values exist in a long period of time and are of larger proportion. Li et al. [47] propose an approach, BoLeRO (Bone Length constrained-based Reconstruction for Occlusion), that focuses on preserving the bone-length constraints in the 3D recovery of the missing joints. They consider the recovery problem a constrained optimization problem with hard and soft constraints. Tan et al. [48] extend the SVT method by grouping it with skeleton constraints, called Skeleton Constrained SVT (SCSVT), that aims to preserve the distances between joints during recovering MoCap data, ensuring that the problem still remains convex. Such methods [47,48] usually employ a specific skeleton model on the basis of the predefined marker set, which ultimately results in high computational complexity. BoLeRO [47] endorses the skeleton constraint which is not only tighter but also needs a much longer processing time as compared to SCSVT [48].

Using the self-similarity method to reconstruct the missing joints is proposed by Aristidou et al. [50] to analyze the motion capture data automatically. A dictionary of motion-words is defined consisting of joint transformations short sequences. To perform comparison of motion-words with K-nearest neighbors, a similarity measure invariant to time and scale has been exploited. This comparison can only be performed between similar kinds of motions. This research work deals only with joint rotation errors while ignoring the possibility of bone-length violations. Moreover, they do not find errors generated through self-collision or the contact failures. If they search for the short motion sequences, they may not be able to find similar KNNs. Several methods deal with the recovery and reconstruction of the human skeleton from noisy and 2D data [17,22,51]. The local models in low-dimensional PCA space can be developed and further optimized by introducing energy constraints [17,22,23].

MoCap data are sequential in nature and very suitable and effective for the recurrent neural models in deep learning. Therefore, several approaches develop various recurrent networks and variants of LSTM [8,34,52,53]. Mall et al. [52] introduce a bidirectional, recurrent neural network architecture for cleaning and refining the MoCap data with noisy and missing joints. It deploys temporal coherence as well as the joint correlations to recover the noisy and incomplete MoCap data. Their approach is a supervised approach, due to which the network must have sufficient data of a new motion type before it can train and learn to clean that motion robustly and reliably. A deep bidirectional attention network [8,53] exploits long-term dependencies, and the attention mechanism is embedded at the encoding and decoding stages in a bidirectional LSTM structure. Cui et al. [54] introduce a feedforward temporal convolutional generative adversarial network (TCGAN) that utilizes hierarchical temporal convolution to model the short-term and reasonable long-term patterns of human motion. Furthermore, they embed the fidelity and consistency discriminators in TCGAN to obtain optimal results. Their method deploys a special preprocessing step of normalization of subjects’ height and scale, in order to reshape the complete mocap data to a uniform height. They further normalize the data by transforming it to in the range of [−1,1]. On the other hand, we do not perform subjects’ height normalization. For an accurate recovery, we assume that the skeleton size of the input query should not be modified and given to the system as it is.

## 3. Methodology

**Figure 1:**
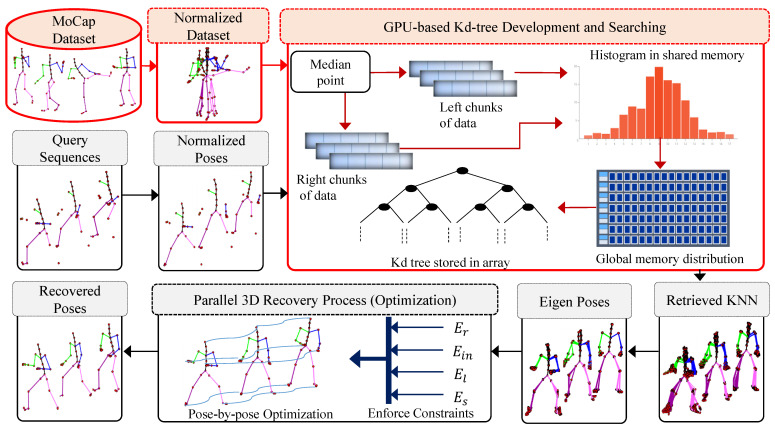
System architecture of the proposed framework. The pre-processing phase is reported in a reddish color while the testing input query phase is depicted in a light gray color.

In this work, we propose a novel data-driven framework to infer corrupted, noisy or missing markers/joints of the MoCap data. We present an automated postprocessing technique to recover the missing markers/joints of the MoCap data, as well as fast searching and robust retrieval of nearest neighbors from large MoCap datasets. In order to exploit the prior information, we first construct a knowledge-base from the existing clean MoCap dataset. To deal with fast searching and retrieving of similar poses from the developed knowledge-base, we propose a parallel architecture to build a *k*d-tree using Compute Unified Device Architecture (CUDA) and the parallel searching strategy of the nearest neighbors from GPU-based *k*d-tree. We also obtain benefits from the CUBLAS library, a GPU-accelerated implementation of the BLAS (Basic Linear Algebra Subroutines) on top of the CUDA, for computations [55]. We query a sequence of input 3D skeleton poses with missing markers/joints to the system to search for nearest neighbors in parallel from the developed knowledge-base. These nearest neighbors are employed further in the recovery process of the missing markers/joints of the MoCap data. For recovery purposes, we present an objective function with four error terms. The objective function is implemented in such a way that we enforce all these four error constraints in a parallel fashion. The optimization process is performed in parallel by using Massive Parallel Architecture (MPA) of GPU. The overall architecture of our proposed framework is shown in Figure 1. We discuss the significance of the proposed parallel approach over the serial method in detail in Section 4.

### 3.1. Problem Formulation

**Figure 2:**
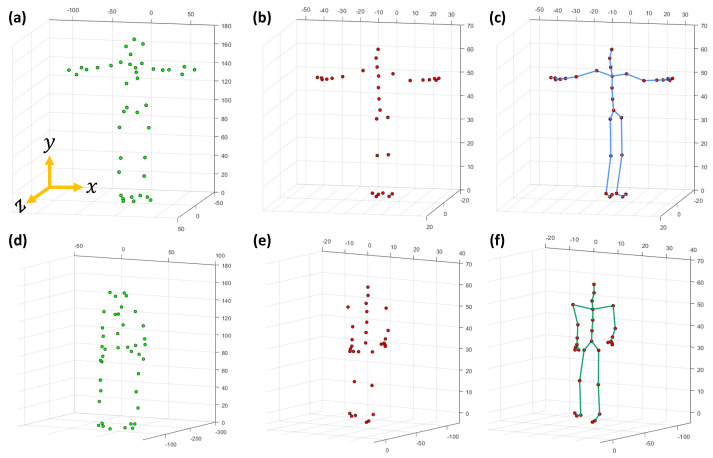
(**a**) The details of the markers (c3d file), (**b**) AMC file, and (**c**) the skeleton (ASF file) of the HDM05 MoCap dataset [9], while (**d**–**f**) elaborate on the details of the marker (c3d file), the AMC file and the skeleton (ASF file) of the CMU MoCap dataset [56], respectively.

**Figure 3:**
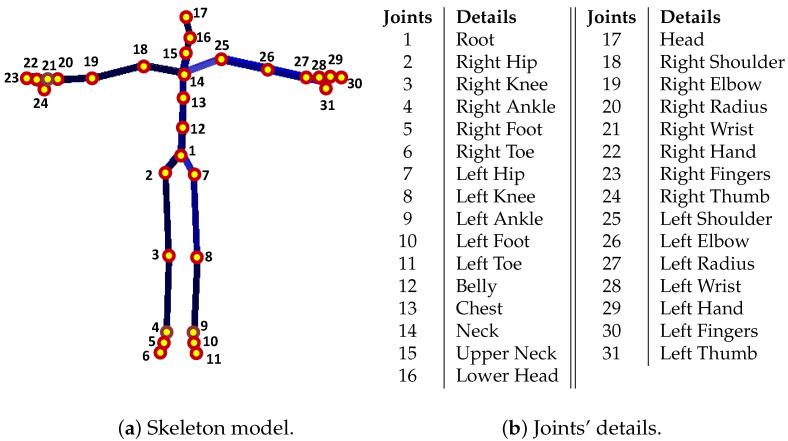
(**a**) The skeleton model P with all joints J=31 for both MoCap dataset, CMU MoCap dataset [56] as well as HDM05 MoCap dataset [9], while (**b**) illustrates all joint details.

First, we describe the notations, symbols, and the representation of the joint-based or marker-based skeleton pose used in the paper. For both the CMU MoCap dataset [56] and the HDM05 MoCap dataset [9], a single joint-based 3D skeleton pose is represented by P that contains N=31 total number of joints. A pose with markers is also expressed by P, but the total number of markers is N=41 in the CMU Mocap dataset [56], while in case of the HDM05 MoCap dataset [9], the total number of markers may vary from 40 to 50. The details of markers, joints, and the skeleton for both the CMU MoCap dataset [56] and the HDM05 MoCap dataset [9] are shown in Figure 2 and Figure 3. Each joint or marker $J\in R^{3}$ has *x*, *y*, and *z* components represented as $j_{x}$, $j_{y}$, and $j_{z}$, respectively. The distance between two adjacent joints is referred to as limb length, and the total number of limbs is denoted as *s*. A joint/marker, e.g., the joint/marker 1, the root joint/marker, is expressed as $J_{1}=\left[j_{1x},j_{1y},j_{1z}\right]$. In this way, a pose becomes $P={\left[J_{1},J_{2},\cdots ,J_{N}\right]}^{T}={\left[j_{1x},j_{1y},j_{1z},j_{2x},j_{2y},j_{2z},\cdots ,j_{Nx},j_{Ny},j_{Nz}\right]}^{T}$, and a motion with *F* number of poses is represented as $\Omega =[P_{1},P_{2},\cdots ,P_{F}]$. ${J_{m}}_{m}$ is the missing joint/marker set, while a number of missing joints or markers is represented with *m*. For instance, a missing joint set with m=6 missing joints is represented as ${J_{m}}_{6}=\left\{J_{9},J_{21},J_{19},J_{5},J_{4},J_{13}\right\}$ or shortly, ${J_{m}}_{6}=\left\{9,21,19,5,4,13\right\}$ with randomly selected missing joints/markers, $J_{9},J_{21},J_{19},J_{5},J_{4},$ and $J_{13}$.

Our proposed framework is subdivided into two major modules. The first module is all about the preprocessing that involves the process of the normalization as well as GPU-based *k*d-tree development. The second module is purely relevant to searching and retrieving nearest neighbors and recovering 3D MoCap data with missing joints or markers. We discuss our proposed framework stepwise as follows.

### 3.2. Normalization

In preprocessing, we normalize our MoCap data first and discard pose variations that are due to the translational or orientation information. Because of translation and/or orientation, the two poses with the same joint angle configurations may have different joint coordinates. In short, we normalize our MoCap data by discarding the translational and orientation information from the MoCap dataset.

#### 3.2.1. Translational Normalization

**Figure 4:**
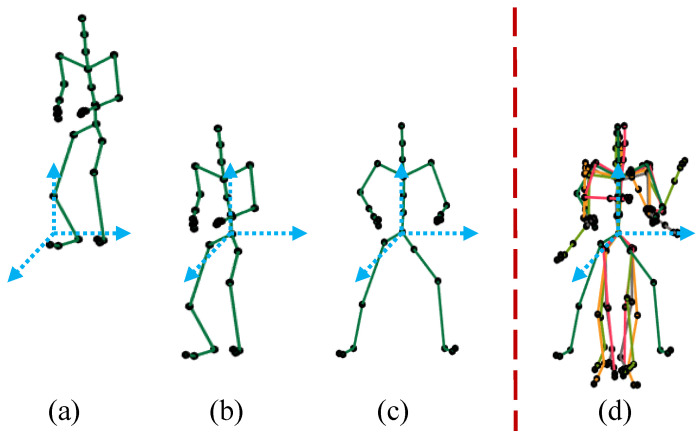
Normalization. (**a**) the original pose; (**b**) the translational normalization; (**c**) orientation normalization; (**d**) multiple normalized poses.

In translational normalization, we translate the pose to its origin axis at the coordinates (0,0,0) in the Euclidean space, as shown in Figure 4b. In fact, we move the root joint to the position at the origin coordinates (0,0,0) and subtract the root joint coordinates of the skeleton from all other joints. In this way, we eliminate the translational information from the pose, and the center of the body mass becomes close to the root joint of the 3D articulated human pose, i.e., the root joint $J_{1}$ of the skeleton is at the origin axis with coordinates (0,0,0). Mathematically,

(1) $$\hat{J_{i}}=J_{i}-J_{1}\;\mathrm{and}\;i\in \left\{1,2,3,\ldots ,N\right\}.$$

#### 3.2.2. Orientational Normalization

In case of orientational normalization, we discard the pose’s orientation by rotating the skeleton along the *y*-axis so that the view of the pose is transformed into a frontal view as shown in Figure 4c. In other words, we rotate all the side-viewed poses with different view angles to just only frontal-viewed poses so that we can avoid any ambiguity that may arise due to the presence of different view angles. In line with [23], all the joints/markers are rotated along the *y*-axis, keeping hip joints’ coordinates parallel to the *z*-axis. For an example, in case of the skeleton with 31 joints, we compute the rotation angle θ at which all the skeleton joints are rotated, using left and right hip joints as,

(2) $$\theta =\arctan\left\{\frac{j_{7x}-j_{2x}}{j_{7z}-j_{2z}}\right\}.$$

Every joint/marker is then rotated by this angle θ, keeping the *y*-axis unchanged,

(3) $${\left[\begin{array}{c} j_{ix}\\ j_{iy}\\ j_{iz}\\ 1 \end{array}\right]}^{T}={\left[\begin{array}{c} j_{ix}\\ j_{iy}\\ j_{iz}\\ 1 \end{array}\right]}^{T}\left[\begin{array}{cccc} \cos(\theta ) & 0 & \sin(\theta ) & 0\\ 0 & 1 & 0 & 0\\ -\sin(\theta ) & 0 & \cos(\theta ) & 0\\ 0 & 0 & 0 & 1 \end{array}\right],$$

where i∈{1,2,3,…,N}.

Finally, we have poses that are entirely free from translational and orientation information. All the poses contain only the knowledge of the performed action. An example of the normalization process is presented in Figure 4. We, after the process of normalization, have only normalized poses in our MoCap dataset, which are the basis for the next step.

### 3.3. Construction of Parallel kd-Tree

We utilize *k*d-tree for KNNs search as a *k*d-tree-based nearest neighbor search together with medium-dimensional feature sets are inordinately practical even for a huge and high-dimensional MoCap datasets [18]. To further speed up the KNNs search, we build a parallel *k*d-tree on the GPU, where the most complex part is sorting the data. In line with [57], we have utilized an off-the-shelf radix sort algorithm. A radix sort algorithm sorts the data by sorting the keys (digits) in two ways i.e., the most significant key and the least significant key. Starting from the least significant key, it sorts a new digit every time and ends up with sorting the whole array. This is the stable sort and can be parallel. The following steps are involved in the parallel radix sort algorithm,

1. The input dataset is divided into multiple subsets, and each subset is assigned to a processor that handles each chunk of data independently.
2. All threads in a block perform the following subsequent tasks for their assigned data;
  - Compute bucket index for all elements in its bucket;
  - Count the number of elements in its bucket;
  - Save this entry histogram of each block in memory;
3. The keys are shifted to the suitable buckets within a processor.

After sorting, the second complicated part of the parallel *k*d-tree is memory access. To keep the implementation simple and efficient, we store the *k*d-tree in the form of arrays, where each node can have two children, and a node at index *i* has its first child at index 2×i and second child at index 2×i+1. The *k*d-tree is constructed by iteratively splitting the data into halves. The median point is used to separate the data into two parts. If the node has the size in the power of 2, we split it using median point, e.g., we split $2^{n}$ elements into two nodes of $2^{n-1}$ elements. For instance, if the node has $2^{4}=16$ elements, it is split into two nodes of size $2^{4-1}=2^{3}=8$ elements. If the node size is less than $2^{n}$, the splitting is performed in such a way that the left child node must be the size of $2^{n-1}$. During the computation of histogram bins, we have used shared memory for fast access, and the execution is performed for one point of data by one thread. The reason behind enforcing the constraint of one data point execution by one thread is to benefit from high-speed atomic operations through local memory. The histogram bins are then copied to the appropriate locations in the global memory.

### 3.4. Nearest Neighbors Search

After building the *k*d-tree, we can use it to search a defined number of K-nearest neighbors from the developed *k*d-tree. For that purpose, we first copy the query frames to GPU memory. To search and find K-nearest neighbors, we efficiently utilize a double-ended queue with K as a limit to store enqueued elements. We do not use stacks and arrays to store the data points as backtracking is involved in searching the nearest neighbors. The basic binary search strategy is adopted to explore similar poses from the *k*d-tree, which is then extended to the parallel architecture. We use Euclidean distance to compare the distance between the reference point in dataset and the query point in order to retain only K-nearest neighbors, which is also computed in parallel by one thread for one query point. We use only the available joints in the query pose to search and retrieve the K-nearest neighbors. Our proposed 3D recovery framework depends upon these retrieved nearest neighbors significantly. The impact of the number of K-nearest neighbors is elaborated in detail in Section 4.

### 3.5. 3D Recovery

To recover the final 3D pose(s), we use the prior domain knowledge available in the motion capture dataset, extracted through the nearest neighbors. To this end, we have K-nearest neighbors in hands for each input pose at time *t*, expressed as $P_{t}=\left\{P_{t,k}|k\;=\;1,\ldots ,K\right\}\in R$. Based on these retrieved K-nearest neighbors, we have developed our local model by applying Principal Component Analysis (PCA). We first compute the 3D local pose model in low-dimensional PCA-based subspace using joint angle configurations of the retrieved KNNs,

(4) $$P_{t}^{r}=\sum_{v=1}^{V}h_{t,v}b_{t,v}+{\hat{P}}_{t},$$

(5) $$P_{t}^{r}=H_{t}B_{t}+{\hat{P}}_{t}.$$

The 3D recovered pose at time *t*, denoted as $P_{t}^{r}$, is the linear combination of a set of *V* number of basis vectors $B_{t}=\left\{b_{t,v}|v=1,\cdots ,V\right\}$ at current time-frame *t* that are basically the principal components (PC) calculated from the KNNs. More precisely, the principal component (PC) coefficients are the eigenvectors having the largest eigenvalues of the covariance matrix of KNNs. The ${\hat{P}}_{t}$ is the mean pose at the current time-frame *t* of the retrieved KNNs. The notation $H_{t}$ is the representation of the current frame with a low dimension in coordinates of PCA space. The number of PC is computed dynamically based on the variance. Next, we design our frame-by-frame objective function, which consists of four error terms, i.e., retrieval error $E_{r}$, input error $E_{in}$, limb length error $E_{l}$, and smoothing error term $E_{s}$. These error terms collectively enforce the local model towards optimal recovered pose(s). Mathematically,

(6) $$\begin{array}{c} E=\underset{P^{r}}{arg min}\left(w_{r}E_{r}+w_{in}E_{in}+w_{l}E_{l}+w_{s}E_{s}\right), \end{array}$$

where the notations $w_{r}$, $w_{in}$, $w_{l}$, and $w_{s}$ denote the weights for the corresponding error terms. These adjacent weights show the significance of each error term. The details about the importance of each error term and an analysis of the contribution of the weight are presented in Section 4.1.5.

#### 3.5.1. Retrieval Error

The retrieval error $E_{r}$ computes the prior likelihood of the current pose and enforces the local pose model according to the pre-existing knowledge available in the MoCap dataset. For that purpose, we utilize Mahalanobis distance,

(7) $$\begin{array}{c} E_{r}={\left[P_{t}^{r}-{\hat{P}}_{t}\right]}^{T}C^{-1}\left[P_{t}^{r}-{\hat{P}}_{t}\right], \end{array}$$

where $P_{t}^{r}$ denotes the recovered pose at frame *t*, ${\hat{P}}_{t}$ represents the mean pose at frame *t*, and $C^{-1}$ is the inverse covariance matrix. The expression ${\left[P_{t}^{r}-{\hat{P}}_{t}\right]}^{T}$ is the transpose of the difference between the mean pose and the recovered pose at frame *t* in PCA space. This term penalizes the deviations of the recovered pose from the retrieved KNNs and therefore bounds the recovered pose implicitly.

#### 3.5.2. Input Error

The input error $E_{in}$ penalizes the deviations between each joint of the input query pose $J_{i,t}^{q}$ and the recovered pose $J_{i,t}^{r}$ as,

(8) $$E_{in}=\frac{1}{\hat{N}}\sum_{i=1}^{\hat{N}}∥J_{i,t}^{r}-J_{i,t}^{q}{∥}^{2}.$$

The input error is only computed for the available number of joints of the corresponding pose, denoted as $\hat{N}$, and $\hat{N}\leq N$.

#### 3.5.3. Limb Length Error

Besides the input error, we also enforce limb length error, where each limb length of the recovered pose $P_{t}^{r}$ is forced to be as close to the limb length of the query pose $P_{t}^{q}$ as possible,

(9) $$E_{l}=\frac{1}{\hat{s}}\sum_{i=1}^{\hat{s}}∥L_{i,t}^{r}-L_{i,t}^{q}{∥}^{2}.$$

where $L_{i,t}^{r}$ and $L_{i,t}^{q}$ are the corresponding limb lengths between two joints of the recovered and query poses respectively at frame *t*. $\hat{s}$ represents the available number of limbs in the input skeleton pose, $\hat{s}\leq s$. This term explicitly enforces anthropometric constraints on the available limbs.

#### 3.5.4. Smoothing Error

The pose reconstruction benefits from enforcing smoothness of the resulting motions. Thus, we add a smoothing error to avoid the jerkiness and jittering artifacts of the high frequency that may arise in the recovered motion,

(10) $$\begin{array}{c} E_{s}=\frac{1}{N}\sum_{i=1}^{N}∥J_{i,t}^{r}-\frac{3J_{i,t-1}^{r}+2J_{i,t-2}^{r}+J_{i,t-3}^{r}}{6}{∥}^{2}, \end{array}$$

where $J_{i,t}^{r}$, $J_{i,t-1}^{r}$, $J_{i,t-2}^{r}$, and $J_{i,t-3}^{r}$ are the $i^{\mathrm{th}}$ joints of the recovered poses at frames *t*, t−1, t−2, and t−3, respectively.

We implement all these error terms in a parallel fashion in CUDA C language on the GPU. For the optimization process, we utilize a nonlinear gradient optimizer, for which the maximum number of function evaluations is set to be 10 K. In contrast, the maximum number of iterations is set to 50 K. The termination tolerance for the function value and a lower bound on the step size are both kept at $1\cdot e^{-4}$.

## 4. Results and Discussion

We evaluate our proposed methodology thoroughly on popular and publicly available benchmark MoCap datasets, CMU [56] and HDM05 [9]. We conduct a bulk of experiments to assess and analyze our proposed framework from various perspectives. In particular, we analyze our approach on different numbers of missing 3D joints/markers: (i) with 3 and 6 numbers of missing joints at different time interval lengths, i.e., 15–120 frames (0.25–2 s) and (ii) with 3/31≈10%, 6/31≈20%, and 9/31≈30% missing markers. We also carried out experiments to see the impact of the GPU parameters, the impact of the retrieved nearest neighbors on the overall system’s performance, and the impact of increasing the missing joints or missing body parts for different motion categories. We compare our proposed approach with other existing competitive state-of-the-art techniques and conclude that our proposed pipeline performs almost outstanding comparatively in terms of accuracy. As mentioned earlier, we evaluate the proposed approach on datasets, CMU [56] and HDM05 [9], and for both datasets, we deploy all the categories of motion sequences that exist in the datasets in order to develop the knowledge-base. We follow, in this paper, two different protocols mentioned in previous works [35,48]:

1. In the **first case,** we deal with the recovery of missing joints [48]. The skeleton model, in this protocol, consists of 31 joints, and the details of the joints are described in Figure 3. In contrast, the details about Acclaim Motion Capture (AMC) file and the Acclaim Skeleton File (ASF) are shown in Figure 2b,c, respectively, for the HDM05 MoCap dataset [9], and for the CMU MoCap dataset [56], the AMC and the ASF files are shown in Figure 2e,f;
2. In the **second case**, in the line of Kucherenko et al. [35], we deal with the recovery of missing markers rather than joints, and the number of markers is N=41 in case of the CMU MoCap dataset [56]. We also assess the performance of our approach with the HDM05 MoCap dataset [9], which contains markers that vary from 40 to 50. The markers (c3d file) for HDM05 and CMU MoCap dataset are shown in Figure 2a,d, respectively.

For both protocols [35,48], we employ Root Mean Squared Error (RMSE) in order to measure the recovery error,

(11) $$\begin{array}{c} Err=\sqrt{\frac{1}{FN}\cdot \sum_{t=1}^{F}\sum_{i=1}^{N}∥J_{t,i}^{r}-J_{t,i}^{g}{∥}^{2}}, \end{array}$$

where $J_{t,i}^{r}$ denotes the $i^{\mathrm{th}}$ joint of the recovered pose $P_{t}^{r}$ at time *t*, while $J_{t,i}^{g}$ means the $i^{\mathrm{th}}$ joint of the ground-truth pose $P_{t}^{g}$ at time *t*.

We first tune the parameters by conducting various experiments that have profound impacts on the performance of our proposed approach. Finally, we thoroughly evaluate the performance of our proposed method by comparing it with other state-of-the-art approaches.

### 4.1. Parameters

We start by conducting preliminary experiments to select and fix the parameters. All these experiments are discussed one by one in the following Subsection.

#### 4.1.1. GPU Memory

First, we carry out experiments to select the type of GPU memory, i.e., global memory or texture memory, for efficient search and retrieval of similar poses. We compare and evaluate both types of memory, especially in terms of elapsed time and memory capacity. For this experiment, we use CMU MoCap datasets with three different sizes, i.e., 45,535, 55,535, and 65,535 number of poses. Furthermore, we conduct this experiment with three different query sizes as well, i.e., 1024, 2048, and 4096 number of poses. We fix the number of nearest neighbors to 64 and then perform searching and retrieval of nearest neighbors.

**Figure 5:**
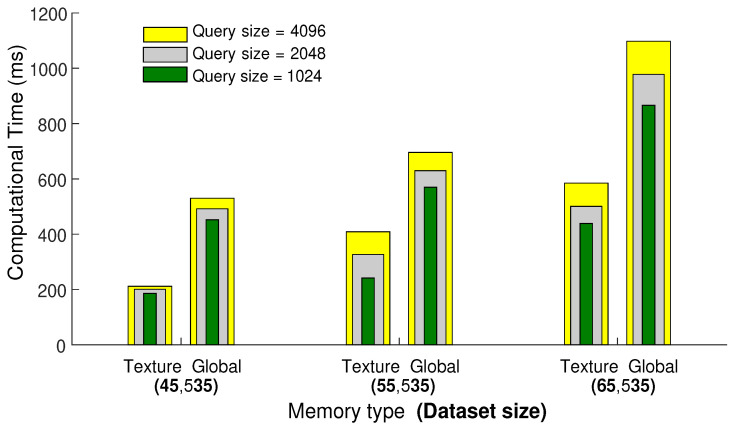
The comparison between the texture and global memories regarding elapsed computational time. For this experiment, KNNs is fixed to be 64, and the computational time is measured in milliseconds.

The results reported in Figure 5 demonstrate that texture memory is fast and efficient compared to global memory, but unfortunately, when we increase the size of the query or dataset, the texture memory goes out of bounds, and the proposed framework halts. There is a trade-off between memory capacity and time complexity. Since we deal with huge and high-dimensional datasets, global memory with large and shared memory capacity is a suitable option for further experiments.

#### 4.1.2. CUBLAS API

**Figure 6:**
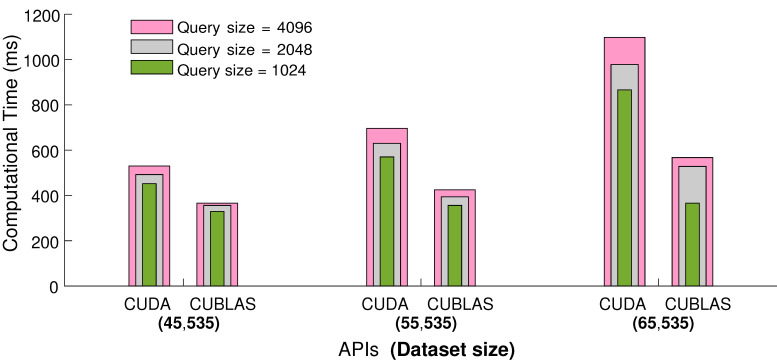
The comparison between CUDA and CUBLAS APIs with respect to elapsed computational time. For this experiment, KNNs are fixed to be 64, and the computational time is measured in milliseconds.

We employ CUBLAS API to enhance and optimize the performance of the process for parallel searching and retrieval of KNNs from the large motion capture datasets. CUBLAS is a library on top of the NVIDIA CUDA, which provides the implementation of basic linear algebra and helps in speeding up the matrices computation. We test CUBLAS API by conducting similar experiments as in the previous Subsection. The results presented in Figure 6 show that, for small sets of query frames, we obtain a 25% speedup in terms of elapsed computational time, and with the increasing number of query frames in input sets, we may achieve up to 50% speedup. Conclusively, we employ global memory and the CUBLAS API for all other experiments.

#### 4.1.3. Nearest Neighbors

**Figure 7:**
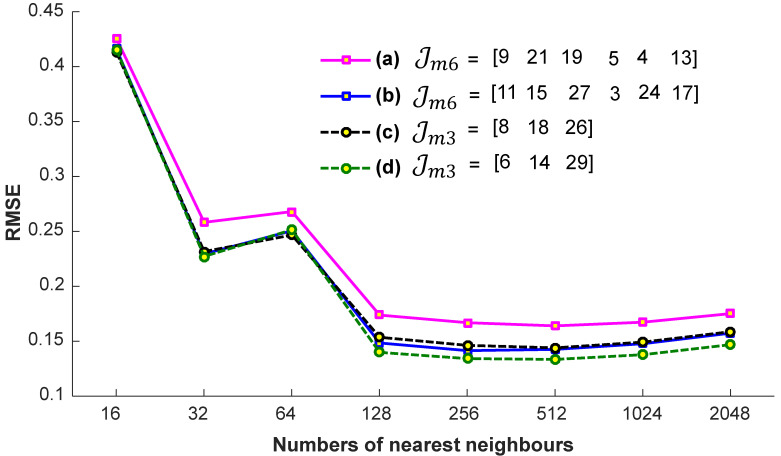
The impact of the number of nearest neighbors in terms of accuracy with different missing joint sets ${J_{m}}_{6}$ and ${J_{m}}_{3}$.

**Figure 8:**
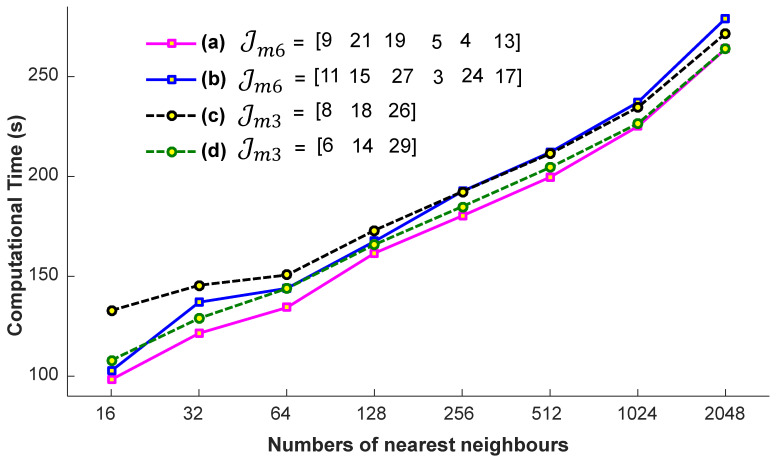
The impact of the number of nearest neighbors in computational time with different missing joint sets ${J_{m}}_{6}$ and ${J_{m}}_{3}$.

We conduct a few preparatory experiments to fix the number of nearest neighbors, denoted as K. We evaluate the efficiency of the proposed system on the account of nearest neighbors, when the input query sequences are with a different missing joint set, ${J_{m}}_{m}$ with m=6 and m=3, i.e., ${J_{m}}_{6}=\left\{9,21,19,5,4,13\right\}$, ${J_{m}}_{6}=\left\{11,15,27,3,24,17\right\}$, ${J_{m}}_{3}=\left\{8,18,26\right\}$, and ${J_{m}}_{3}=\left\{6,14,29\right\}$. These missing joints are selected randomly. For these experiments, the values engaged for K are 32, 64, 128, 256, and 512. We conclude from the results reported in Figure 7 that we achieve almost the best results at a particular number of the nearest neighbors, i.e., K =256. If we increase the number of nearest neighbors, the accuracy remains nearly the same. We also see the impact of K-nearest neighbors in terms of computational time. From the results presented in Figure 8, it is quite obvious that the time increases linearly with the increase in the number of nearest neighbors. Generally, the selection of the suitable number of nearest neighbors is a trade-off between the accuracy and the computational time. There is no doubt that, if there is a smaller number of nearest neighbors, the system will be faster, but we have to compromise the accuracy then. In short, in this paper, the best suitable value for K is 256, and we fix this value for the rest of the paper, independent of the datasets.

#### 4.1.4. Principal Components

**Figure 9:**
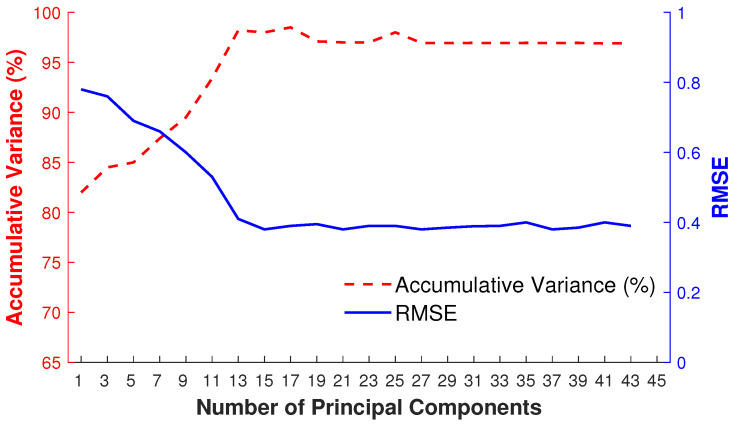
Accumulative variance (%) and RMSE for the dynamic selection of the number of principal components.

We apply PCA to predict the local linear model in a low-dimensional linear subspace based on the retrieved nearest neighbors. To fix the number of principal components (eigenposes), we adopt a frame-by-frame adaptive and dynamic approach that basically depends on the captured variance of K retrieved nearest neighbors of the given input query pose. Therefore, as a result, the number of principal components varies for each input query pose. We demonstrate the impact of the number of principal components (eigenposes) by an example depicted in Figure 9 that visualizes the accumulative variance and the RMSE obtained for a different number of eigenposes for K retrieved nearest neighbors of the given input query pose. We can observe that, at a certain number of eigenposes, e.g., at 15 eigenposes in this case, the error becomes minimum with the highest accumulative variance. By increasing the number of eigenposes, the accumulative variance remains stable.

**Figure 10:**
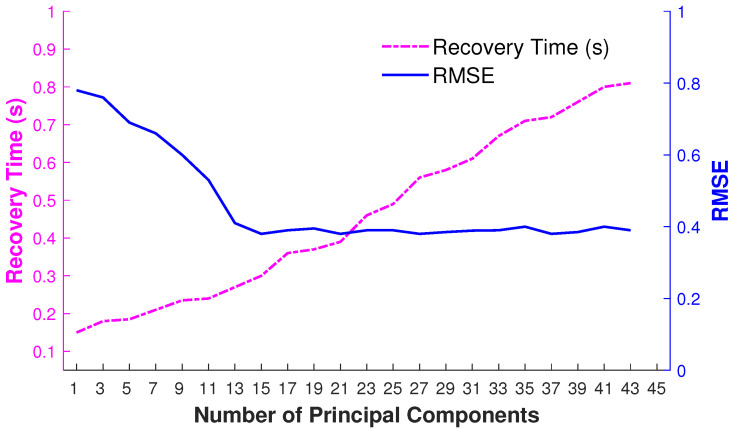
RMSE and the recovery time in seconds for the dynamic selection of the number of principal components.

We have also observed that error optimization is much faster in a lower-dimensional PCA space through experimentations. Therefore, it is worth having the overhead of computing principal components for each input query pose. The findings about recovery time and RMSE are reported in Figure 10.

#### 4.1.5. Impact of Error Terms

**Figure 11:**
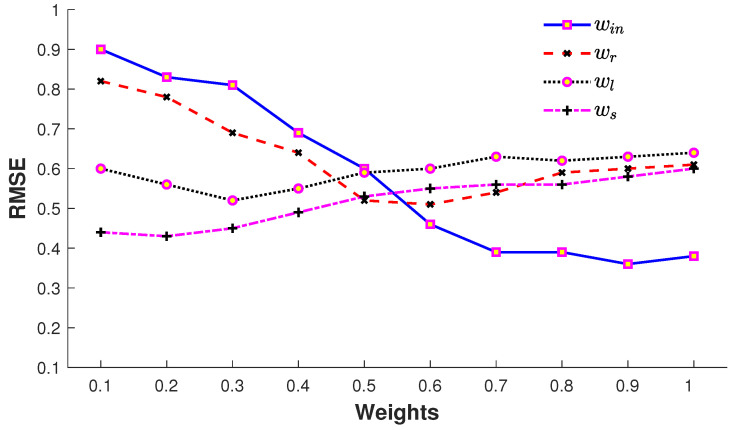
Impact of weights $w_{r},w_{in}$, $w_{l}$, and $w_{s}$ used in (6).

The impact of error terms with their corresponding weights in Equation (6) is elaborated in Figure 11. The input error term $E_{in}$ contributes substantially as expected since this error term endorses the recovered pose according to the input query pose with missing joints. It is also observed that, with an increase in the number of missing joints, the impact of this error term is reduced correspondingly. The error term $E_{r}$ executes the pre-existing knowledge of the MoCap dataset and also plays a vital role in the 3D recovery process. The retrieval error term (7) is biased towards larger joint sets since the pose variation of the retrieved poses is larger for smaller joint sets, while the input error term $E_{in}$ (8) is biased towards a smaller number of joints. The trade-off between $E_{in}$ (8) and $E_{r}$ (7) is steered by the weights $w_{r}$ and $w_{in}$ in (6). The error terms, $E_{l}$ (9) and $E_{s}$ (10), do not result in a large drop of the error, but they refine and smoothen the 3D recovered pose. We fix all these weights as $w_{r}=0.55,w_{in}=0.9$, $w_{l}=0.3$, and $w_{s}=0.15$ in our experiments.

### 4.2. Comparison with State-of-the-Art Methods

This section evaluates our proposed approach by comparing it with other state-of-the-art techniques in terms of 3D recovery of missing joints and markers, respectively.

#### 4.2.1. Missing Joints

In the first experiment, we follow the same protocol as mentioned by Tan et al. [48] and deploy the same MoCap sequences of CMU [56], where each pose consists of 31 joints. Similarly, we first partition the MoCap sequences into different time intervals, where the interval length varies, i.e., 15–120 frames (0.25–2 s). We then randomly remove a fixed number of joints from the MoCap sequence for each time interval. The number of joints removed randomly defines the *error rate* of the MoCap sequence with missing or corrupted joints. For example, a 3/31≈10% error rate means that a skeleton pose contains three corrupted/missing joints, and a 6/31≈20% error rate means that a skeleton pose includes six corrupted/missing joints. We then compare our approach with other state-of-the-art methods [46,47,48] with different time intervals while the number of missing joints are kept at m=3 and m=6. The results reported in Table 1 show that our proposed approach almost outperforms all other state-of-the-art methods for most of the motion categories. In very few cases such as *kicking* and *jumping*, our proposed method does not provide extraordinary results and produces a slightly higher error as compared to other competing state-of-the-art approaches [47,48]. However, the results of our approach are still competitive as the gap is not too high.

We compute the mean values for all time intervals with both number of missing joints m=3 and m=6. We also compute the mean values for all types of action sequences in case of every state-of-the-art method. The results are reported in Table 1 which shows that our proposed approach outpaces all the state-of-the-art techniques in almost every scenario of mean computations.

Generally, RMSE increases when the number of missing joints increases. For example, with m=6 missing joints, the error increases for all the time intervals and motion categories. Furthermore, RMSE also increases with the increase in time interval length. At the highest time interval length, e.g., l=120, the error becomes highest. In a few cases, the error reduces when the time interval length increases, e.g., for motion category *jumptwist*, the error reduces when time interval length jumps from l=60 to l=90. This is not as surprising since the input query data are different for different time intervals. Due to this, the retrieved nearest neighbors and the results for the final 3D recovery are different as well. It might be possible that a few certain skeleton poses, having some vital information, do not exist in the motion sequences generated with time interval length l=60 but are available in the case of interval length l=90.

We have also conducted a statistical analysis of the results of all the state-of-the-art methods presented in Table 1. We performed the ANOVA (Analysis of Variance) tests separately when the missing joints are m=3 and m=6. With m=3 missing joints, we obtain the *F*-statistic (the ratio of mean squared errors) as 2.15, and the *p*-value (probability) equals 0.1165. Thus, the null hypothesis (the mean results of all the reported methods are equal) is not rejected. In other words, this test indicates that the mean results of all methods including our approach are not statistically significantly different from one another. With m=6 missing joints, the *F*-statistic is 4.0 and the *p*-value is 0.0173, which is less than α=0.05. It indicates that the null hypothesis is rejected, and a statistically significant difference exists between the results of at least two methods. Based on a more detailed statistical analysis, we found that the reported state-of-the-art methods, such as SVT [46], SCSVT [48] and BoLeRO [47], have no statistically significantly different results from each other, but our proposed method significantly differs from BSVT [46], and in comparison to SCSVT [48] and BoLeRO [47], the mean results of our approach do not differ from them significantly. However, in both cases with the missing joints m=3 and m=6, our approach results in a lower recovery error (RMSE) compared to any other state-of-the-art approach as reported in Table 1.

#### 4.2.2. Missing Markers

We compare our methodology to other state-of-the-art approaches with a different protocol in the second experiment. We follow the protocol of Kucherenko et al. [35] and work with the same CMU MoCap sequences of *basketball*, *jump turn*, and *boxing*, where each pose consists of 41 markers. We employ RMSE again to measure the recovery error. We conduct this experiment with 10%, 20%, and 30% missing markers. The results reported in Table 2 show that, for all cases of missing markers, our approach again executes the outstanding results compared to all other existing state-of-the-art techniques [32,35,39], interpolation method, and the method based on PCA with 18 principal components. Notably, the standard interpolation method also performs well, but it is suitable for the shorter gaps (less than 0.1 sec) only. In our case, if a few specific joints are missing continuously in an input motion sequence, our proposed scheme does not have as much impact on the 3D recovery results since our approach is a frame-by-frame method and relies entirely on the available joints of the input motion sequence.

We also test our approach on the HDM05 MoCap dataset [9]. We train our model on the HDM05 MoCap dataset and use the same CMU MoCap sequences for testing. As a result, error increases, but our approach still executes outstanding performance compared to other state-of-the-art methods for all motion categories.

We also compute the mean values for all types of action sequences for every method with 10%, 20%, and 30% missing markers. The results are reported in Table 2, which interprets that our proposed approach evidently performs better as compared to all the competing state-of-the-art approaches in every scenario of mean computations.

Similar to the statistical analysis performed for the values presented in Table 1, we also conduct a statistical analysis of the results of all the state-of-the-art techniques for the missing markers reported in Table 2. With 10% missing markers, we obtain the *F*-statistic 3.21, and the *p*-value equals 0.025, (<α=0.05). Thus, at least two of the compared methods differ from each other significantly. A more detailed statistical analysis shows that all of the reported state-of-the-art approaches do not have a significant difference from each other, but our approach is significantly different from Burke and Lasenby [39]. With 20% missing markers, the *F*-statistic and *p*-value are 1.48 and 0.244, respectively, which indicates that there is no significant difference between the mean results of all the state-of-the-art methods including our approach. With 30% missing markers, the *F*-statistic and *p*-value are 1.83 and 0.150, respectively, which shows that the mean results of all the methods are not significantly different from each other. Nevertheless, in all cases of 10%, 20%, and 30% missing markers, the results reported in Table 2 show that our approach results in lower recovery error (RMSE) compared to any other reported state-of-the-art approach.

#### 4.2.3. Parallel vs. Serial

**Figure 12:**
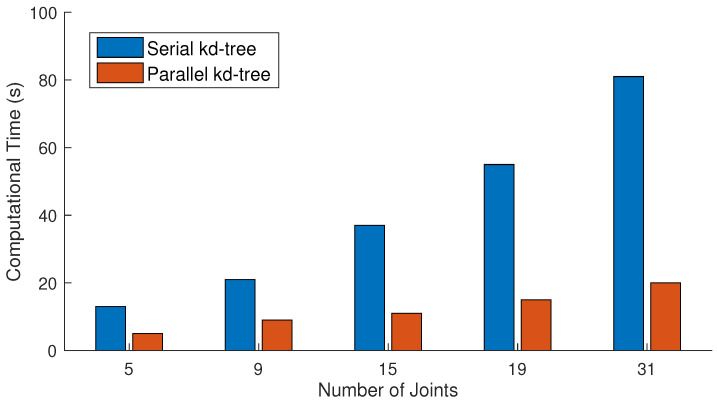
Comparative analysis of serial and parallel construction of *k*d-tree when the sizes of dataset and query are 65,535 and 2048, respectively. The number of nearest neighbors is fixed to 64 for these experiments.

**Figure 13:**
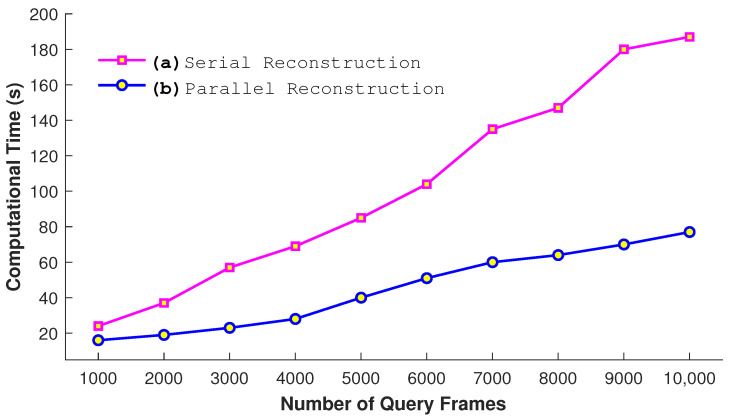
Comparative analysis of serial and parallel reconstruction with a different number of query frames when K=256.

We also perform experiments to justify our parallel approach. We conduct a comparison between the serial and parallel construction of *k*d-tree when the size of the dataset is 65,535 frames and the query consists of 2048 frames. The number of nearest neighbors is fixed to 64 for this experiments. The results presented in Figure 12 show that the parallel construction of *k*d-tree takes very few seconds in comparison to the serial approach as expected. Similarly, another experiment is conducted to see the impact of the parallel reconstruction. In this experiment, we utilize a different number of query frames as input. From the results in Figure 13, it is quite obvious that the process of the parallel reconstruction is significantly fast as compared to the serial reconstruction. With the larger number of query frames, the difference of time between both increases substantially.

### 4.3. Controlled Experiments

We perform a few controlled experiments in order to see the overall impact of missing joints and missing whole body parts on the performance of our proposed approach. We discuss these experiments as follows.

#### 4.3.1. Impact of Missing Joints

**Figure 14:**
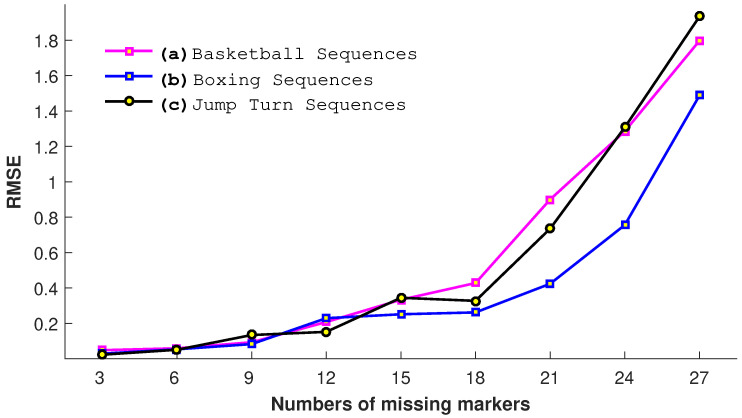
The impact of the number of missing joints in terms of RMSE.

In this experiment, we evaluate our proposed approach with a different number of missing joints *m*, i.e., 3, 6, 9, 12, 15, 18, 21, 24, and 27. We conduct this experiment on three motion categories: *basketball*, *jump turn*, and *boxing*. The results shown in Figure 14 elaborate that, as expected, the error increases with the increase in the number of missing joints. The highest error is found when the input query poses have 27 missing joints. The results show that, when the number of missing joints cross some specific value, i.e., 18, the RMSE increases exponentially for all three motion categories. We may conclude from this experiment that our proposed approach is reasonably able to endure up to 14–18 missing joints.

#### 4.3.2. Impact of Missing Body Parts

**Figure 15:**
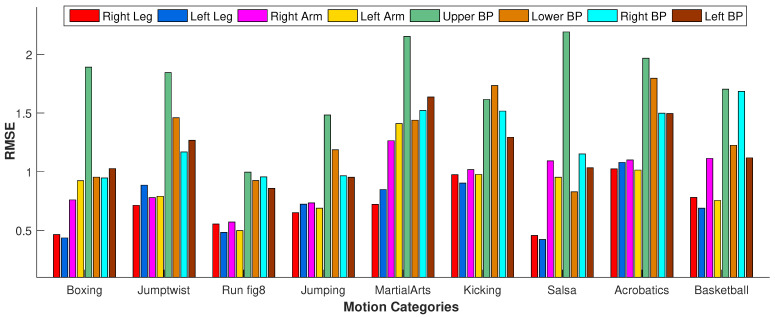
The impact of the missing joints of the different body parts regarding RMSE.

In the second experiment, instead of selecting missing joints randomly, we drop out all the joints of some specific body parts. We test our approach when all the markers of the following body parts are dropped out: *right leg*, *left leg*, *right arm*, *left arm*, *upper body part*, *lower body part*, *right body part*, and *left body part*. We perform this experiment on various motion classes such as *boxing*, *jumptwist*, *run fig8*, *jumping*, *martial arts*, *kicking*, *salsa*, *acrobats*, *basketball*, etc. We observe from the results shown in Figure 15 that RMSE is at its peak when the *upper body part* is missing. This is because, for most of the motions, the upper body joints contribute significantly to performing actions compared to the other body joints. However, in a few motion categories such as *run fig8* and *kicking*, RMSE with missing *upper body parts* is less than different motion categories such as *boxing*, *jumptwist*, *martial arts*, etc., because the upper body joints do not contribute as much to the motion classes as mentioned earlier. Conclusively, RMSE increases if the joints of the most contributing body parts in performing some particular motion are missing.

### 4.4. Qualitative Evaluation

We also evaluate our proposed methodology qualitatively. For this purpose, we conduct experiments in two different directions.

**Figure 16:**
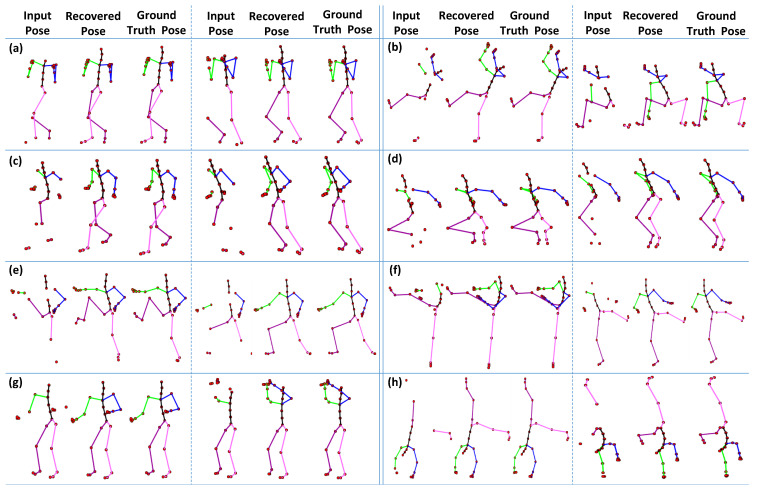
The 3D recovery results for different motion categories, when the number of missing joints is m=6. (**a**) Boxing; (**b**) Jumptwist; (**c**) Run fig8; (**d**) Jumping; (**e**) Martial Arts; (**f**) Kicking; (**g**) Salsa; (**h**) Acrobat.

In the first part of the experiment, we fix the size of the missing joints as equal to 6, which are selected randomly for different types of motion categories, i.e., *boxing*, *jumptwist*, *run fig8*, *jumping*, *martial arts*, *kicking*, *salsa*, and *acrobat*, etc. We compare the 3D recovery results of the proposed approach to ground-truth poses. The results reported in Figure 16 show that our proposed method executes significantly good results compared to the ground-truth poses for almost all motion categories.

**Figure 17:**
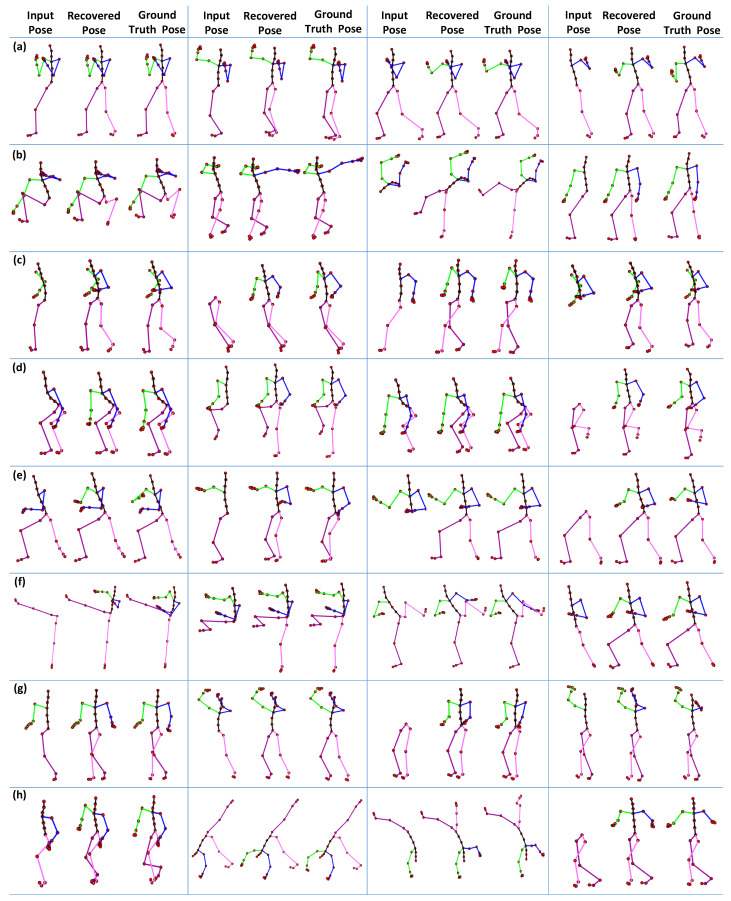
The 3D recovery results for different motion categories when missing a whole body part. (**a**) Boxing; (**b**) Jumptwist; (**c**) Run fig8; (**d**) Jumping; (**e**) Martial Arts; (**f**) Kicking; (**g**) Salsa; (**h**) Acrobat.

In the second part of the experiment, we drop all markers of the different body parts (see details about body parts in Section 4.3.2) and then evaluate the proposed methodology qualitatively. We conduct this experiment on the same motion categories, as mentioned earlier in the first part of the experiment. As shown in Figure 17, the results are pretty remarkable. Even with the missing joints of the complete body parts, our proposed methodology still recovers the poses efficiently.

## 5. Limitations

Our framework depends on MoCap datasets forming the knowledge-base. If a particular motion class is missing from the knowledge-base, the retrieval step will find incorrect samples and thus not yield good results.

**Figure 18:**
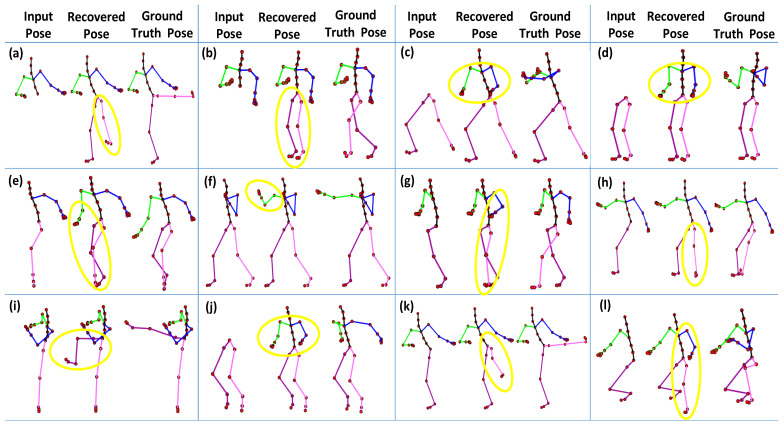
A few worst or failure cases of 3D recovery when all the joints of a whole body part are missing. (**a**) Kicking (all the joints of the *lower body part* are missing); (**b**) Acrobat (*lower body part*); (**c**) Martial arts (*upper body part*); (**d**) Salsa (*upper body part*); (**e**) Basketball (*right body part*); (**f**) Boxing (*right arm*); (**g**) Run fig8 (*left body part*); (**h**) Jumptwist (*left leg*); (**i**) Kicking (*right leg*); (**j**) Jumping (*upper body part*); (**k**) Kicking (*left leg*); (**l**) Martial arts (*right body part*). The yellow circles point towards the incorrect recovery of missing joints.

A few body parts play a substantial role in performing some motions. The significance of the body parts depends upon the types of activities. For example, the body parts, i.e., *right arm*, *left arm*, and the *upper body part*, are significant in *boxing* motion, but the *right leg* and the *left leg* do not contribute a lot to performing that motion. Similarly, the *upper body part* is noteworthy in the *jumping* motion, and the *lower body part* is vital for *kicking* motion. In short, if the missing markers belong to the aforementioned body parts, our approach may produce higher recovery error, as apparent in Figure 15. Furthermore, a few qualitative examples of the failure cases are reported in Figure 18.

## 6. Conclusions and Future Work

We have proposed a novel approach for recovering 3D human motion capture data with missing joints or markers. We normalize the 3D human poses and build a knowledge-base using these normalized 3D poses. We develop a parallel *k*d-tree on GPU for fast search and efficient retrieving of the nearest neighbors from MoCap data for the input query poses. The retrieved nearest neighbors are employed to predict a prior local model, refined further by introducing the optimization function with multiple error terms. We perform all these steps in parallel on the GPU, and as a result, we make the proposed framework significantly fast. We have conducted extensive quantitative and qualitative experiments on publicly available benchmark MoCap datasets. We conclude from the results that our proposed methodology results in lower RMSE compared to almost all other state-of-the-art approaches with 10%, 20%, and 30% missing joints or markers. Our approach also works well, even though the joints of whole body parts are missing. Moreover, our experiments demonstrate that the proposed framework achieves outstanding results even when the training data are from a different MoCap dataset.

The proposed method focuses on the recovery of missing joints of a single person in a scene, and may be extended to the recovery of the missing joints of multiple persons simultaneously, interacting with each other in a scene. Furthermore, the proposed approach might be shifted to video-based scenarios where the query is in 2D with some missing skeleton information. Another essential direction for future work might be integrating the recovery of missing joints with action recognition and person identification.

## Figures and Tables

**Table 1:** Comparison with other state-of-the-art methods in terms of RMSE of the recovered MoCap sequences.

Sequences	Method	m=3						m=6					
		l=15	l=30	l=60	l=90	l=120	Mean	l=15	l=30	l=60	l=90	l=120	Mean
	BSVT [46]	0.052	0.051	0.053	0.056	0.061	0.055 ± 0.004	0.098	0.118	0.138	0.128	0.140	0.124 ± 0.017
14_01	SCSVT [48]	0.046	0.043	0.045	**0.045**	**0.048**	0.045 ± 0.002	0.085	0.104	0.118	0.102	0.105	0.103 ± 0.012
(Boxing)	BoLeRO [47]	0.068	0.066	0.068	0.063	0.072	0.067 ± 0.003	0.100	0.112	0.143	0.112	0.130	0.119 ± 0.017
	Our	**0.025**	**0.027**	**0.043**	0.061	0.063	**0.044 ± 0.018**	**0.054**	**0.054**	**0.065**	**0.076**	**0.080**	**0.066 ± 0.012**
	BSVT [46]	0.281	0.613	1.563	2.038	2.499	1.399 ± 0.937	0.716	1.119	2.204	3.060	3.745	2.169 ± 1.274
85_02	SCSVT [48]	0.120	0.191	0.230	0.204	0.249	0.199 ± 0.050	0.248	0.315	0.354	0.485	0.321	0.344 ± 0.087
(Jumptwist)	BoLeRO [47]	0.145	0.154	0.214	0.266	0.326	0.221 ± 0.076	0.290	0.340	0.366	0.538	0.397	0.386 ± 0.094
	Our	**0.042**	**0.027**	**0.044**	**0.010**	**0.011**	**0.027 ± 0.016**	**0.121**	**0.091**	**0.031**	**0.026**	**0.030**	**0.060 ± 0.044**
	BSVT [46]	0.075	0.136	0.510	0.380	1.261	0.472 ± 0.475	0.156	0.340	1.148	1.634	3.280	1.312 ± 1.253
143_04	SCSVT [48]	0.059	0.088	0.124	0.128	0.155	0.111 ± 0.038	0.117	0.193	0.220	0.270	0.290	0.218 ± 0.068
(Run fig 8)	BoLeRO [47]	0.057	0.062	0.155	0.135	0.141	0.110 ± 0.047	0.105	0.143	0.174	0.314	0.403	0.228 ± 0.126
	Our	**0.039**	**0.038**	**0.037**	**0.050**	**0.051**	**0.043 ± 0.007**	**0.050**	**0.049**	**0.058**	**0.073**	**0.069**	**0.060 ± 0.011**
	BSVT [46]	0.046	0.065	0.111	0.091	0.238	0.110 ± 0.076	0.128	0.182	0.239	0.255	0.423	0.245 ± 0.111
49_02	SCSVT [48]	**0.039**	**0.053**	0.086	0.068	0.093	0.068 ± 0.022	0.119	0.140	0.132	0.146	0.153	0.138 ± 0.013
(Jumping)	BoLeRO [47]	0.057	0.058	**0.074**	**0.064**	**0.079**	**0.066 ± 0.010**	**0.103**	**0.099**	0.139	0.133	0.233	0.141 ± 0.054
	Our	0.056	0.062	0.095	0.111	0.130	0.091 ± 0.032	0.130	0.139	**0.131**	**0.131**	**0.151**	**0.136 ± 0.009**
	BSVT [46]	0.097	0.120	0.168	0.166	0.209	0.152 ± 0.044	0.201	0.246	0.404	0.428	0.453	0.346 ± 0.115
135_02	SCSVT [48]	0.083	0.096	0.139	**0.131**	**0.151**	**0.120 ± 0.029**	0.181	0.211	0.333	0.247	**0.280**	0.250 ± 0.059
(Martial Arts)	BoLeRO [47]	0.138	0.144	0.144	0.161	0.195	0.156 ± 0.023	0.216	0.228	0.287	0.266	0.282	0.256 ± 0.032
	Our	**0.073**	**0.086**	**0.129**	0.175	0.219	0.136 ± 0.061	**0.112**	**0.133**	**0.167**	**0.204**	0.283	**0.180 ± 0.067**
	BSVT [46]	0.057	0.071	0.144	0.261	0.288	0.164 ± 0.106	0.132	0.180	0.326	0.500	0.608	0.349 ± 0.204
135_11	SCSVT [48]	**0.052**	0.065	0.095	**0.095**	0.104	0.082 ± 0.022	0.125	0.150	**0.184**	0.160	0.157	**0.155 ± 0.021**
(Kicking)	BoLeRO [47]	0.054	**0.058**	**0.081**	0.099	**0.074**	**0.073 ± 0.018**	0.092	**0.102**	0.383	**0.154**	**0.114**	0.169 ± 0.122
	Our	0.071	0.112	0.165	0.258	0.302	0.182 ± 0.097	**0.083**	0.124	0.240	0.265	0.341	0.210 ± 0.106
	BSVT [46]	0.070	0.086	0.102	0.167	0.174	0.120 ± 0.048	0.159	0.206	0.233	0.390	0.344	0.266 ± 0.097
61_05	SCSVT [48]	0.060	0.063	0.080	0.100	0.102	0.081 ± 0.020	0.137	0.168	0.144	0.185	0.179	0.163 ± 0.021
(Salsa)	BoLeRO [47]	0.073	0.085	0.086	0.100	0.112	0.091 ± 0.015	0.123	0.145	0.156	0.151	0.209	0.157 ± 0.032
	Our	**0.042**	**0.048**	**0.054**	**0.084**	**0.100**	**0.066 ± 0.025**	**0.068**	**0.067**	**0.086**	**0.107**	**0.121**	**0.090 ± 0.024**
	BSVT [46]	0.131	0.202	0.274	0.342	0.347	0.259 ± 0.093	0.363	0.617	0.576	1.036	0.949	0.708 ± 0.279
88_04	SCSVT [48]	0.101	0.123	**0.146**	**0.147**	**0.141**	**0.132 ± 0.020**	0.265	0.432	0.317	0.314	**0.261**	0.318 ± 0.070
(Acrobatics)	BoLeRO [47]	0.155	0.143	0.201	0.178	0.177	0.171 ± 0.023	0.270	0.286	0.341	0.302	0.297	0.299 ± 0.026
	Our	**0.090**	**0.103**	0.148	0.211	0.244	0.159 ± 0.067	**0.152**	**0.170**	**0.205**	**0.232**	0.333	**0.218 ± 0.071**
**Mean**	BSVT [46] ${}^{‡}$	0.101	0.179	0.366	0.438	0.635	0.344 ± 0.212	0.244	0.376	0.659	0.929	1.243	0.690 ± 0.407
	SCSVT [48]	0.070	0.090	0.118	**0.115**	**0.130**	0.105 ± 0.024	0.160	0.214	0.225	0.239	0.218	0.211 ± 0.032
	BoLeRO [47]	0.093	0.096	0.128	0.133	0.147	0.119 ± 0.024	0.162	0.182	0.249	0.246	0.258	0.229 ± 0.044
	Our ${}^{‡}$	**0.055**	**0.063**	**0.089**	0.120	0.140	**0.093 ± 0.036**	**0.096**	**0.103**	**0.123**	**0.139**	**0.176**	**0.128 ± 0.032**

BSVT [46] is Basic Singular Value Thresholding, SCSVT [48] is Skeleton Constrained Singular Value Thresholding, and BoLeRO [47] is Bone Length constrained-based Reconstruction for Occlusion. The lowest error is shown in bold. ${}^{‡}$ The methods are statistically significantly different from each other as the *p*-value <0.05, when the missing joints are m=6.

**Table 2:** Comparison with other state-of-the-art approaches in terms of RMSE over the missing markers of the recovered MoCap sequences. Ours-1 (training: CMU, testing: CMU), Ours-2 (training: HDM05, testing: CMU).

(a) 10% Missing Markers					
**Method**	**Training Dataset**	**Motion Sequences**			**Mean**
		**BasketBall**	**Boxing**	**Jump Turn**	
PCA (PC-18)	CMU	4.75±1.50	5.29±1.48	4.84±3.40	4.96±2.13
PCA (PC-18)	HDM05	4.87±1.63	5.49±1.51	4.97±3.51	5.11±2.22
Interpolation *	CMU	0.64±0.03	1.06±0.12	1.74±0.3	1.15±0.15
Peng et al. [32] *	CMU	n.a.	n.a.	n.a.	n.a.
Burke and Lasenby[39] *${}^{‡}$	CMU	4.56±0.17	3.47±0.19	15.97±1.34	8.00±0.57
Kucherenko et al. [35] (Window)	CMU	2.34±0.27	2.61±0.21	4.40±0.5	3.12±0.33
Kucherenko et al. [35] (LSTM)	CMU	1.21±0.02	1.44±0.02	2.52±0.3	1.72±0.11
Ours-1 ${}^{‡}$	CMU	0.55±0.22	0.7±0.16	0.62±0.27	0.62±0.22
Ours-2	HDM05	1.14±0.82	1.11±0.50	1.28±1.16	1.18±0.83
**(b) 20% Missing Markers**					
**Method**	**Training Dataset**	**Motion Sequences**			**Mean**
		**BasketBall**	**Boxing**	**Jump Turn**	
PCA (PC-18)	CMU	4.75±1.68	5.32±1.39	4.85±3.41	4.96±2.16
PCA (PC-18)	HDM05	4.89±1.89	5.51±1.52	4.99±3.53	5.13±2.31
Interpolation *	CMU	0.67±0.04	1.09±0.07	1.91±0.31	1.22±0.14
Peng et al. [32] *	CMU	n.a.	4.94	5.12	5.03
Burke and Lasenby [39] *	CMU	4.18±0.48	3.98±0.07	27.1±1.21	11.75±0.59
Kucherenko et al. [35] (Window)	CMU	2.42±0.32	2.77±0.13	4.30±0.75	3.16±0.40
Kucherenko et al. [35] (LSTM)	CMU	1.34±0.01	1.58±0.04	2.67±0.2	1.86±0.08
Ours-1	CMU	0.63±0.30	0.84±0.37	0.81±0.61	0.76±0.43
Ours-2	HDM05	1.20±1.91	1.17±1.10	1.36±1.34	1.24±1.45
**(c) 30% Missing Markers**					
**Method**	**Training Dataset**	**Motion Sequences**			**Mean**
		**BasketBall**	**Boxing**	**Jump Turn**	
PCA (PC-18)	CMU	4.76±1.36	5.31±1.33	4.89±3.50	4.99±2.06
PCA (PC-18)	HDM05	4.90±1.91	5.53±1.55	5.01±3.53	5.15±2.33
Interpolation *	CMU	0.7±0.1	1.21±0.14	2.29±0.3	1.40±0.18
Peng et al. [32] *	CMU	n.a.	4.36	4.9	4.63
Burke and Lasenby [39] *	CMU	4.23±0.57	4.01±0.26	34.9±2.55	14.38±1.13
Kucherenko et al. [35] (Window)	CMU	2.33±0.13	2.63±0.08	4.53±0.48	3.16±0.23
Kucherenko et al. [35] (LSTM)	CMU	1.48±0.03	1.75±0.07	3.1±0.25	2.11±0.12
Ours-1	CMU	0.81±0.58	0.97±0.7	1.03±1.51	0.94±0.93
Ours-2	HDM05	1.32±1.77	1.21±1.38	1.39±1.87	1.31±1.67
**(d) Mean**					
**Method**	**Training Dataset**	**Motion Sequences**			**Mean**
		**BasketBall**	**Boxing**	**Jump Turn**	
PCA (PC-18)	CMU	4.75±1.51	5.31±1.40	4.86±3.44	4.97±2.12
PCA (PC-18)	HDM05	4.89±1.81	5.51±1.53	4.99±3.52	5.13±2.29
Interpolation	CMU	0.67±0.06	1.12±0.11	1.98±0.30	1.26±0.16
Peng et al. [32]	CMU	n.a.	4.65	5.01	4.83
Burke and Lasenby [39]	CMU	4.32±0.41	3.82±0.17	25.99±1.70	11.38±0.76
Kucherenko et al. [35] (Window)	CMU	2.36±0.24	2.67±0.14	4.41±0.58	3.15±0.32
Kucherenko et al. [35] (LSTM)	CMU	1.34±0.02	1.59±0.04	2.76±0.25	1.90±0.10
Ours-1	CMU	0.66±0.37	0.84±0.41	0.82±0.80	0.77±0.53
Ours-2	HDM05	1.22±1.5	1.16±0.99	1.34±1.46	1.24±1.32

* The numbers are extracted from [35]. The lowest error is shown in bold. ${}^{‡}$ The methods are statistically significantly different from each other as the *p*-value <0.05.

## Data Availability

Not applicable.
